# Reply to: Letter to the Editor of Journal of Otolaryngology regarding “Risk of diabetes in patients with sleep apnea: comparison of surgery versus CPAP in a long-term follow-up study”

**DOI:** 10.1186/s40463-023-00684-z

**Published:** 2023-12-01

**Authors:** Carlos O’Connor Reina, Laura Rodriguez Alcala, Jose Maria Ignacio, María Teresa Garcia Iriarte, Marina Carrasco Llatas, Juan Carlos Casado Morente, David Perez del Rey, Irene Marbán Alvarez, Gema Marbán Ibarburu, Peter Baptista, Guillermo Plaza

**Affiliations:** 1 Otorhinolaryngology Department, Hospital Quironsalud Marbella, 29680 Marbella, Spain; 2 Otorhinolaryngology Department, Hospital Quironsalud Campo de Gibraltar, Palmones, Spain; 3 Neumology Department, Hospital Quironsalud Marbella, Marbella, Spain; 4 Otorhinolaryngology Department, Hospital Virgen de Valme, Seville, Spain; 5 Otorhinolaryngology Department Hospital Dr Peset, Valencia, Spain; 6 Biomedical Informatics Group, Universidad Politécnica de Madrid, Madrid, Spain; 7 Otorhinolaryngology Department, Clínica Universitaria de Navarra, Pamplona, Spain; 8 Otorhinolaryngology Department, Hospital Universitario de Fuenlabrada, Universidad Rey Juan Carlos, Madrid, Spain; 9 Otorhinolaryngology Department, Hospital Sanitas La Zarzuela, Madrid, Spain

**Keywords:** Sleep apnea, Diabetes, Big data, Upper airway surgery

## Abstract

A recent Letter published, in the *Journal of Otolaryngology—Head & Neck Surgery* in response to our original article “Risk of diabetes in patients with sleep apnea: comparison of surgery versus Continous Positive Airway Pressure in a long-term follow-up study” raised some issues we would like to address here. However, we thank the authors for their effort and time in analyzing our manuscript and we want to facilitate a balanced discussion on this topic with our reply.

## Selection bias

Truong et al. [1] suggested that our findings were confounded by a selection bias. The cohorts of the study were balanced using propensity score matching. Multiple comorbidities were selected to be balanced; before running the analysis, the two cohorts were matched by selectively removing individuals to render the differences for the selected comorbidities statistically nonsignificant. The selection of comorbidities to balance was based on the typical comorbidities of obstructive sleep apnea (OSA) patients reported in the literature [2, 3]. However, it is impossible to prove empirically that a full set of cofounders has been included in the propensity score matching model [4], especially in a retrospective real-world data study. We considered that it was also essential to avoid adding too many cofounders to the propensity score matching model, which could have led to “overfitting” the data and a decrease in the population representativeness of the sample [5]. Only patients with data going back at least three months before the procedure were included in the electronic health records to minimize bias due to data incompleteness.

The study’s index event was the initiation of continuous positive airway pressure (CPAP)/surgery after OSA diagnosis, and the 5-year follow-up also included the beginning of treatment.

## Coding and cohort queries

We excluded patients with cancer or those undergoing cancer treatment to ensure that the OSA was not caused by cancer/treatment. We excluded patients younger than 18 years of age. There is no code for UAS to treat sleep-disordered breathing. In CPAP, we also considered the CPT code 94660 to include ambulatory CPAP patients in the cohort. The SNOMED-CT code 47545007 and HCPCS A7034 were not included, but the number of patients with data for both was residual compared with the ICD and CPT codes.

## OSA treatment efficacy and TriNetX limitations

It is not possible to identify adherence rates to CPAP using TriNetX. However, to date, no scientific randomized clinical trial has shown an association between diabetes and adherence to CPAP [6]. All published studies are based on the good glycemic control obtained when CPAP is used, but disease prevention is not addressed.

## Updated risk of diabetes methods and results

We thank Truong et al. [1] for their effort in recalculating the study. They rebuilt the study, and their conclusions are basically the same as those in the original study, which is reassuring. We are grateful that our findings were subjected to their different approach to using this methodology.

## Conclusions

Based on exploiting big data with two different methodologies, we conclude that UAS is more effective in preventing diabetes than CPAP.

## Data Availability

The data that support the findings of this study are available from Trinetx but restrictions apply to the availability of these data, which were used under license for the current study, and so are not publicly available. Data are however available from the authors upon reasonable request and with permission of Trinetx.

## Abbreviations

OSA: Obstructive sleep apnea
CPAP: Continuous positive airway pressure
UAS: Upper airway surgery
CPT: Current procedural terminology
SNOMED: Systematized nomenclature of medicine
ICD-10-PCS: International classification of diseases-10 procedure coding system
